# Survival following primary androgen deprivation therapy among men with localized prostate cancer

**Published:** 2008

**Authors:** Pawan Vasudeva, Apul Goel, Divakar Dalela

**Affiliations:** Department of Urology, C.S.M.M.U (Upgraded King George Medical College), Lucknow, Uttar Pradesh, India

## SUMMARY

This population-based cohort study evaluated the impact of primary androgen deprivation therapy (PADT) on cancer-specific and overall survival in elderly men with localized (T1-2) prostate cancer.

Data of 89,877 men > 66 years with T1-2 prostate cancer diagnosed from 1992-2002 was obtained from the population-based Surveillance, Epidemiology, and End Results program (SEER) database. Exclusion criteria included men who either received definitive local therapy (e.g., prostatectomy or radiation) within 180 days of diagnosis or had died during the same period, those with missing data or unknown cancer grade and those in whom ADT was started before cancer diagnosis. This left a group of 19,271 men of median age 77 years available for analysis; 7867 (41%) of these had received PADT in the form of luteinizing hormone-releasing hormone agonists or B/L orchiectomy as primary cancer therapy during the first 180 days following diagnosis whereas 11,404 (59%) had been conservatively managed during this period.

Overall and cancer-specific survival was analyzed in these two groups using instrument variable analysis (IVA) technique. The instrumental variables used for the two treatment groups, i.e. PADT and conservative management were high PADT use area and low PADT use area, respectively. These corresponded to the top and bottom tertiles of PADT utilization areas which were calculated from the proportion of patients receiving PADT in each health service area.

There were 11,045 deaths from all causes in the study group of which 1560 were prostate cancer-related deaths (median follow-up for overall survival being 81 months).

Using IVA, patients in the PADT group had lower 10-year prostate cancer-specific survival (80.1% vs. 82.6%; hazard ratio: 1.17) and no increase in 10-year overall survival (30.2% vs. 30.3%; hazard ratio: 1.00) when compared with patients on conservative management. IVA in the subset of patients with moderately differentiated cancer (Gleason score 5-7) again revealed significantly poorer prostate cancer-specific survival in the PADT group as compared to the conservative management group (*P* value: < 0.001) though no difference was seen in the overall survival. However, similar analysis done in the subset of patients with poorly differentiated cancer (Gleason score 8-10) showed a borderline improvement in cancer-specific survival in the PADT group as compared to the conservative management group (*P* value: 0.49) though again no significant difference was seen in the overall survival.

In patients receiving PADT and surviving at least three years (5826 patients), cancer-specific and overall mortality was lower in those receiving therapy for < 12 months as compared to those receiving therapy for longer periods (13-36 months), irrespective of grade of tumor.

Based on the above findings, the authors concluded that PADT is not associated with improved survival when compared with conservative management in the majority of elderly men with localized cancer prostate.

## COMMENTS

Although PADT for localized cancer prostate is not advocated as standard therapy by major guidelines,[1–3] its use among clinicians as an alternative to surgery, radiation and conservative management is on the rise,[4] especially in elderly men suffering from the disease. Though there have been encouraging results in the use of ADT as an adjunct to surgery/ radiation in men with localized disease,[5] data on its use as primary therapy is lacking. Analysis of the data in this study showed that contrary to the expectation of a possible benefit, instituting PADT in fact had an adverse impact on cancer-specific mortality without any overall survival benefit in the PADT group as a whole and also in the subset with moderately differentiated cancers. Only in the subgroup with poorly differentiated cancers did instituting PADT show some benefit as far as cancer-specific morality was concerned, though again no survival benefit was noted.

A plausible explanation for the findings may be that when well/ moderately differentiated cells, which may never have harmed the patients overall survival are suppressed by instituting ADT, rapidly growing malignant clones may establish and overgrow. This would then increase the probability of death due to prostate cancer instead of a competing cause of death.

In the light of the above findings, the fact that more data on PADT use in this setting is currently lacking and the increasing knowledge that ADT may be associated not only with impotency, gynecomastia, hot flashes etc., but also may increase the risk of fracture, diabetes mellitus, coronary heart disease and sudden cardiac death,[6] the clinician should carefully consider the rationale for initiating PADT in elderly men with localized cancer prostate.
